# Study on Gas Sorption and Iodine Uptake of a Metal-Organic Framework Based on Curcumin

**DOI:** 10.3390/molecules28135237

**Published:** 2023-07-06

**Authors:** Hongmin Su, Yang Zhou, Tao Huang, Fuxing Sun

**Affiliations:** 1 School of Environmental Science and Engineering, Yancheng Institute of Technology, Yancheng 224051, China; suhm@ycit.edu.cn (H.S.); zy1057401716@163.com (Y.Z.); ht18262570262@126.com (T.H.); 2 State Key Laboratory of Inorganic Synthesis and Preparative Chemistry, College of Chemistry, Jilin University, Changchun 130021, China

**Keywords:** Metal-Organic framework, gas sorption, iodine uptake

## Abstract

Medi-MOF-1 is a highly porous Metal-Organic framework (MOF) constructed from Zn(II) and curcumin. The obtained crystal was characterized using powder X-ray diffraction (PXRD) and scanning electron microscopy (SEM). A micrometer-sized crystal with similar morphology was successfully obtained using the solvothermal method. Thanks to its high surface area, good stability, and abound pores, the as-synthesized medi-MOF-1 could be used as a functional porous material to adsorb different gases (H_2_, CO_2_, CH_4_, and N_2_) and iodine (I_2_). The activated sample exhibited a high I_2_ adsorption ability of 1.936 g g^–1^ at room temperature via vapor diffusion. Meanwhile, the adsorbed I_2_ could be released slowly in ethanol, confirming the potential application for I_2_ adsorption.

## 1. Introduction

With the rapid growth of global energy demand, the pursuit of energy storage technology has drawn special attention from scientists in recent years [1,2]. Compared to coal and petroleum, gaseous fuels are more friendly to the environment. However, the transportation and storage of gaseous fuels is a major challenge for researchers. Therefore, functional porous materials to capture or separate gas have been investigated, such as zeolite [3,4,5], Metal-Organic frameworks [6,7,8], and porous organic polymers [9,10]. Among these materials, Metal-Organic frameworks (MOFs), constructed from secondary building units (SBUs) and organic linkers, have been widely researched in the past decades. Due to their high porosities, tunable pore sizes, etc., the unique structural advantage of MOFs has been widely pursued for gas storage and separation [11,12,13,14], catalysis [15], chemical sensing [16,17,18], and so on. Hydrogen is considered as one of the alternative energy sources for fossil fuels. With a large surface area and tunable pore structure, MOFs can also provide unsaturated metal sites for hydrogen sorption [19,20,21]. At present, growing efforts to develop MOFs that efficiently capture or separate CO_2_ is desired [22,23,24,25]. Compared to traditional methods, MOFs based on adsorption to capture or separate CO_2_ can reduce energy consumption, showing great advantages in these technologies. Thus, constructing an economical and preferable tunable pore structure and pores is highly desirable.

Moreover, MOFs also exhibit potential applications for toxic waste elimination, such as heavy metal [26], uranium [27], iodine [28,29,30,31], and so on. Radioactive iodine ^129^I and ^131^I have a long half-life (1.57 × 10^7^ years), which compounds damage the environment and human beings. How to dispose the nuclear waste timely and effectively has become an important issue that needs to be addressed. Although zerovalent iron [32], zeolite [33], and functionalized clays [34] have been used for radioiodine capture, their low absorption capacity and less interactive sites with iodine have limited the applications. Thus, MOFs with designable architecture and excellent properties have been synthesized for iodine capture. There are two kinds of MOFs for iodine adsorption, including non-iodine MOFs and iodine-templated MOFs [35,36,37]. The presence of iodide groups in the framework can affect the iodine uptake [38,39]. For example, [(ZnI_2_)_3_(tpt)_2_] was constructed with ZnI_2_ nodes with tpt (2,4,6-tris(4-pyridyl)-1,3,5-triazine), which has a good I_2_ loading amount of 173 wt.% at room temperature, which is similar to reported for Cu-BTC (175 wt.%) [40]. In addition, non-iodine MOFs have been developed and utilized in the field to capture I_2_. [Zr_6_O_4_(OH)_4_(edb)_6_]_n_ can uptake iodine by means of chemi- and physisorption [41]. Although the presence of the iodide group in the framework can affect the iodine uptake, the structure of the organic ligands is too complex, which can make it difficult to obtain in the synthetic reaction. Hence, choosing a simple ligand and reaction process to construct porous MOFs for gas and I_2_ sorption is highly needed.

**Scheme 1:**
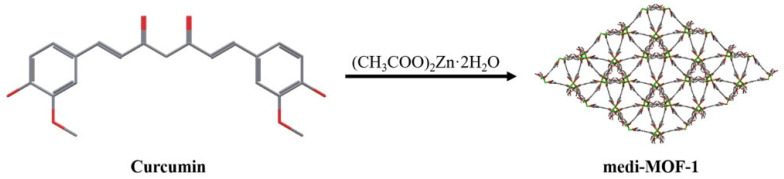
The synthetic process of medi-MOF-1.

In our previous work [42], medi-MOF-1 was successfully synthesized using structurally symmetric ligand curcumin with the Zn(II) ion. In this study, we report on micrometer-sized porous medi-MOF-1 via large-scale reactions (Scheme 1). Subsequently, we studied the adsorption properties of medi-MOF-1 for H_2_, CO_2_, CH_4_, and N_2_. It is revealed that medi-MOF-1 can adsorb 1.57 wt.% H_2_ at 77 K and 1 bar, and displayed commendable CO_2_ adsorption and selectivity for CO_2_ over CH_4_ and N_2_ at 273 K. Furthermore, it is worth noting that medi-MOF-1 exhibits an outstanding I_2_ adsorption capacity of 1.936 g g^−1^. It is also revealed that the I_2_ sorption process of medi-MOF-1 is reversible.

## 2. Results

### 2.1. Physicochemical Properties of Medi-MOF-1

**Figure 1:**
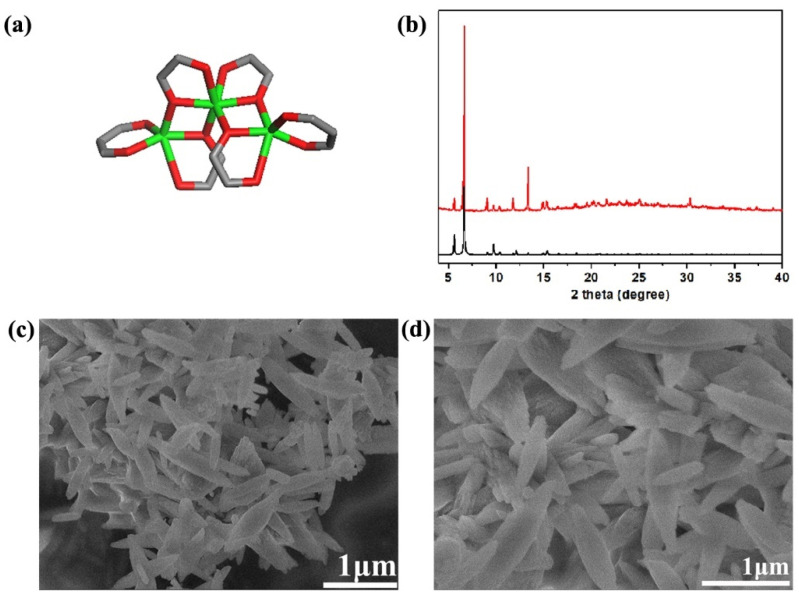
(**a**) Trinuclear Zn(II) clusters of medi-MOF-1 (Zn, green; C, gray; O, red; H are omitted for clarity.); (**b**) PXRD patterns of medi-MOF-1 (black, simulated; red, as-synthesized medi-MOF-1); (**c**,**d**) SEM images of the as-synthesized medi-MOF-1.

As reported previously by us, medi-MOF-1 crystallizes in the trigonal chiral space group *P*3_2_21. It is constructed by trinuclear clusters and curcumin ligands, leading to a three-dimensional (3D) porous coordination framework (Scheme 1). In this work, we synthesized medi-MOF-1 by expanding the reaction by 10 times. Powder X-ray diffraction (PXRD) of medi-MOF-1 confirmed the phase purity of the bulk crystalline materials due to the same PXRD pattern with the simulated data (Figure 1b). The pore structure properties of medi-MOF-1 were characterized at 77 K. The N_2_ adsorption–desorption isotherm of type-I adsorption curves with a capillary in the low P/P_0_ region. It confirms that the as-synthesized medi-MOF-1 is microporous. The adsorption isotherm data were fitted to the Langmuir equation and gave a surface area of 563 m^2^ g^−1^ (BET surface area: 475 m^2^ g^−1^). This surface area is similar to nanosized medi-MOF-1 particles but far below the value of 2675 m^2^ g^−1^ reported large size of the crystal [42]. The decreased size and crystallinity may be the main reason for reducing the BET of medi-MOF-1 [43]. Subsequently, scanning electron microscopy (SEM) technologies have been widely used to study the morphology of nanoparticles. Herein, SEM images were recorded for inspecting the morphology and structure of as-synthesized medi-MOF-1. As seen in Figure 1c,d, SEM images show that the prepared solid samples are agglomerated with small nanocrystals which have good uniformity and dimensional consistency. SEM images showed rod-like crystals of medi-MOF-1 with a diameter of 0.2 μm and length of 1 μm which had a similar morphology to that of larger crystals [42]. According to these results, the as-synthesized medi-MOF-1 can be successfully synthesized as crystalline powder materials.

### 2.2. Gas Sorption Properties

**Figure 2:**
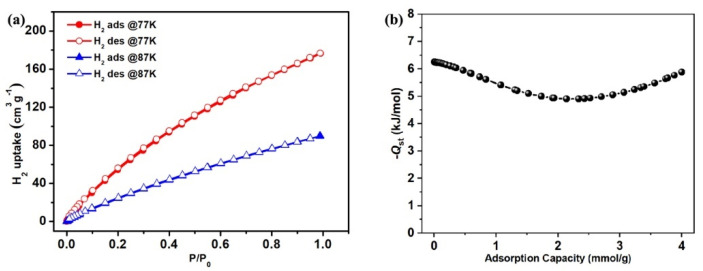
(**a**) Hydrogen adsorption and desorption isotherms of medi-MOF-1 (red circle at 77 K, blue triangle at 87 K); (**b**) the calculated Q_st_ plots for H_2_ uptake in the medi-MOF-1.

Much effort has been devoted to hydrogen storage since hydrogen is considered to be an excellent alternative energy source. In order to test the hydrogen uptake of medi-MOF-1, the as-synthesized medi-MOF-1 was activated after soaking in the solvent of CH_2_Cl_2_ for 2 days [44] and heating at 100 °C under vacuum for 8 h. The H_2_ sorption experiments of activated samples were measured, which showed that the H_2_ uptake of medi-MOF-1 is as high as 1.57 wt.% at 77 K and 1 atm (Figure 2a), which is similar to bio-MOF-11 constructed from biomolecules solely [45]. We calculated the isosteric heats of adsorption (*Q*_st_) of H_2_ using the Clausius equation following the fitting of the isotherm data at 77 and 87 K using a virial equation. The initial *Q*_st_ value for H_2_ of medi-MOF-1 is calculated to be −6.25 kJ mol^−1^ at zero coverage (Figure 2b). Furthermore, the initial *Q*_st_ value for H_2_ of medi-MOF-1 is smaller than that of bio-MOF-11 (−13 kJ mol^−1^) [46,47]. These results may be attributed to the more metal clusters of bio-MOF-11, for the force of metal clusters on hydrogen is greater than that of the benzene ring on hydrogen. Additionally, the M_2_(olz) materials are bioactive frameworks with similar frameworks exhibiting potential H_2_ storage capacities [48]. The relevant BET data and H_2_ adsorption capacities of the selected MOFs constructed with biomolecules or drug molecules are summarized and tabulated in Table S1. The isosteric heat of Zn_2_(olz) adsorption is similar to medi-MOF-1, which is lower than other M_2_(olz) frameworks in the series due to fewer open metal sites in the activated materials.

**Figure 3:**
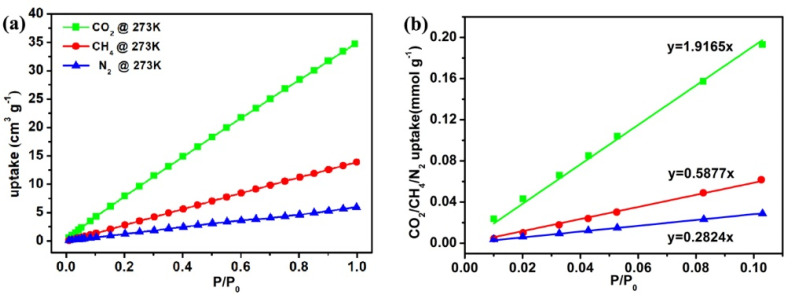
(**a**) CO_2_, CH_4_, and N_2_ adsorption isotherms at 273 K for medi-MOF-1; (**b**) CO_2_/CH_4_/N_2_ selectivity for medi-MOF-1 (273 K) calculated using the Henry’s law constants in the linear low pressure (<0.1 bar) range.

As we know, the separation of CO_2_ to CH_4_ and N_2_ by porous materials is favorable in the environment. In this text, we measured the adsorption ability of different gases in medi-MOF-1. At 273 K and 1 bar, medi-MOF-1 exhibits higher uptake of CO_2_ than CH_4_ and N_2_—which is 34.7 cm^3^ g^−1^ (1.55 mmol g^−1^). The datapoint is lower than those of bio-MOFs constructed with biomolecules [49]. The reason for this phenomenon may be the fewer adsorption sites in the framework of medi-MOF-1. The relevant BET data and CO_2_ adsorption capacities of the selected bio-MOFs are summarized and tabulated in Table 1. The maximum uptakes of CH_4_ and N_2_ are 13.9 cm^3^ g^−1^ and 5.95 cm^3^ g^−1^ at 273 K and 1 bar, respectively (Figure 3a), which are lower than that of CO_2_ in the same conditions. Especially, the uptake of CO_2_ for medi-MOF-1 is 2.5 times as large as CH_4_ and 5.8 times as large as that of N_2_. The adsorption selectivity of CO_2_ relative to CH_4_ and N_2_ was calculated using Henry’s law before 0.1 bar. Based on Henry’s law, the material shows CO_2_ over N_2_ or CH_4_ adsorption selectivity (Figure 3b), ranking medi-MOF-1 as better porous adsorbents constructed from biomolecules for separating CO_2_ from N_2_ [50,51,52].

### 2.3. Iodine Uptake and Release

**Figure 4:**
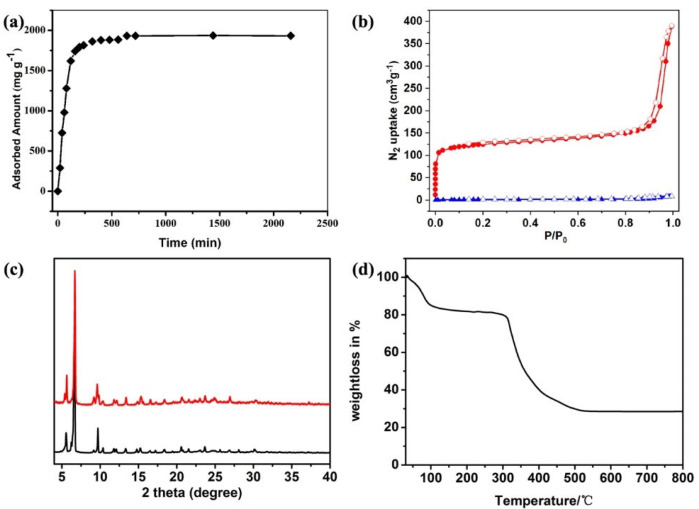
(**a**) Iodine vapor adsorption curves at 298 K under ambient pressure for medi-MOF-1; (**b**) N_2_ adsorption and desorption isotherms of activated medi-MOF-1 (red), I_2_@medi-MOF-1 (blue).; (**c**) PXRD patterns of activated medi-MOF-1(black), and I_2_@medi-MOF-1 (red); (**d**) the TGA curve of I_2_@medi-MOF-1.

To prevent the presence of radioactive toxic gases in the environment, researchers have focused on the preparation of selective porous materials. As medi-MOF-1 has developed porosity and a stable crystalline structure, the I_2_ uptake experiment was carried out in the vapor phase. Before the adsorption experiments were started, the sample was activated at 60 °C for 6 h under a vacuum. Iodine uptake was measured using the gravimetric method. In the vapor phase, 30 mg of medi-MOF-1 [39] was placed in an I_2_ chamber at 298 K. After 12 h, the color of medi-MOF-1 changed from orange-red to dark brown. No further change was observed after 10 h, and the maximum adsorbed amount of I_2_ was as high as 1.936 g g^−1^ (Figure 4a). Compared to other typically porous MOFs usually used for I_2_ adsorption via vapor diffusion, medi-MOF-1 also exhibits higher I_2_ adsorption. The relevant BET data and iodine adsorption capacities of the selected MOFs are summarized and tabulated in Table 2. This result may be attributed to the frameworks with conjugated π-electrons, which could produce multiple interactions for iodine [54,55]. Iodine templates are introduced into the assembly process of MOFs such as (ZnI_2_)_3_(tpt)_2_ at room temperature in order to affect the uptake capacities of iodine [56]. Owing to medi-MOF-1 can keep its crystal structure unchanged in ethanol, we soaked 100 mg of medi-MOF-1 crystals in 3 mL of a dry ethanol solution of I_2_ in a sealed glass vial at room temperature. After 48 h, I_2_ molecules were mostly adsorbed by the free active sites in medi-MOF-1, and no more free sites were left. There is almost no N_2_ sorption in the low-pressure region after the incorporation of iodine, indicating that I_2_ completely fills the pores (Figure 4b). A PXRD study was carried out before and after the I_2_ absorption experiment and proved that the framework of medi-MOF-1 retained the host framework crystallinity after loading the I_2_ molecules (Figure 4c). Thermogravimetric analysis (TGA) was performed to check the I_2_ loading amount. The I_2_@MOF showed a weight loss of ~52% from 100 to 500 °C (Figure 4d). Combined with the thermogravimetric curve and the molecular formula of medi-MOF-1, it can be calculated as 500 mg of iodine per gram of MOF. Compared to other porous MOFs usually used for I_2_ adsorption via solution-based processes in ethanol, medi-MOF-1 also exhibits higher iodine adsorption than JLU-Liu14 [57,58]. The high loading of I_2_ for medi-MOF-1 can be attributed to the different pore sizes of these MOFs and the possible interaction between the porous skeleton and I_2_.

**Figure 5:**
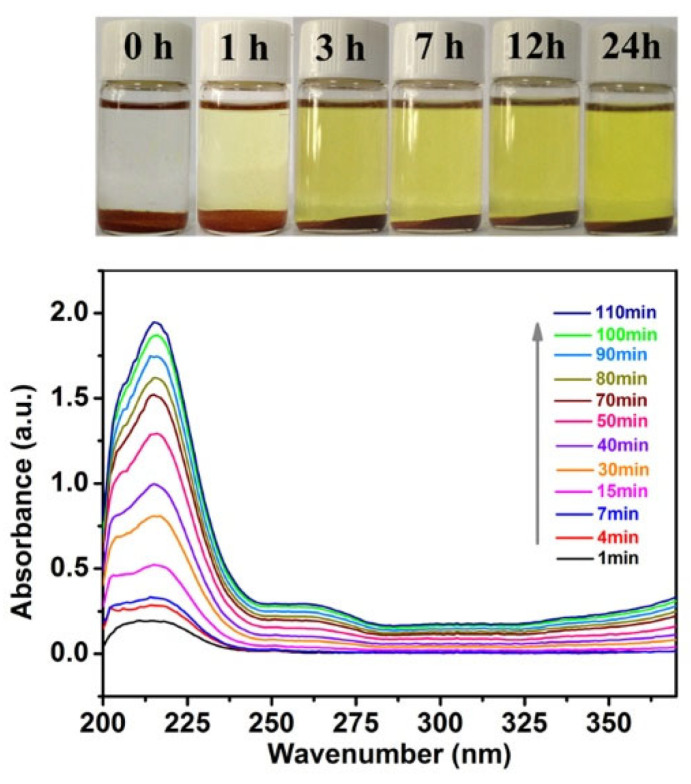
Up: Photographs of the time-dependent I_2_ desorption process by medi-MOF-1 in ethanol; below: UV-vis spectra for I_2_ release from medi-MOF-1 in ethanol.

From the point of view of recyclability, the I_2_ desorption from the porous framework is also essential. The I_2_ release process was detected by UV-visible (UV-vis) spectra. The captured I_2_ could be easily separated from the frameworks upon immersion in I_2_-loaded medi-MOF-1 in ethanol. A UV-vis spectrophotometer recorded the release of I_2_ from the medi-MOF-1 framework at different times at room temperature. When the I_2_@medi-MOF-1 was soaked in dry ethanol, the color of the iodine-loaded sample changed gradually from dark brown to orange-red. Afterwards, the release slows down and subsequently, the color of the ethanol solution changes from colorless to yellow. As illustrated in Figure 5, UV-vis spectra show absorption bands at λ_max_ = 218 and 263 nm, which can be attributed to I_2_. And the band observed at 263 nm may be assigned to polyiodide ions (I_3_^−^), established due to the reaction of I_2_ with decomposed iodide. The release of iodine increased with time, suggesting that this desorption behavior of iodine is based on host–guest interactions. This adsorption and release of I_2_ by medi-MOF-1 reveals that the I_2_ sorption process of the MOF is reversible.

## 3. Materials and Methods

### 3.1. Materials and General Methods

All reagents were obtained from commercial sources and used as received. All the other chemical reagents used were of AR grade. PXRD was collected on a Rigaku D/Max 2550 X-ray diffractometer with Cu-Kα radiation (λ = 1.5418 Å). FT-IR spectra were obtained by a Nicolet Impact 410 Fourier-transform infrared spectrometer in the 400–4000 cm^−1^ range with KBr pellets. TGA was performed under an air atmosphere in the range of 30–800 °C at a heating rate of 10 °C min^−1^ using a Perkin-Elmer TGA 7 thermogravimetric analyzer. Gas adsorption and desorption isotherms were measured on Quantachrom Autosorb-iQ after degassing the sample for 8 h at 100 °C. H_2_ adsorption tests were performed at 77 and 87 K. CO_2_, CH_4_, and N_2_ adsorption tests were performed at 273 K. The morphologies of the powder materials were recorded using a JEOL-JSM-6700F field-emission scanning electron microscope (SEM).

### 3.2. Synthesis of Medi-MOF-1 and Iodine-Loaded Medi-MOF-1

Medi-MOF-1 have been produced on a large scale. A mixture of Zn(OAc)_2_·2H_2_O (200 mg, 0.9112 mmol), curcumin (600 mg, 1.6287 mmol), N,N-dimethylformamide (40 mL), and ethanol (10 mL) was sealed into a 100 mL capped vessel. The vessel was heated at 75 °C. The crystals were obtained after 5 days and washed with DMF. Yield: 55% (based on curcumin). Iodine uptake experiment was carried out via vapor diffusion and solution phase. A 100 mg sample was immersed in an ethanol solution of iodine. The complete absorption experiment was done at room temperature for 48 h.

## 4. Conclusions

In summary, we report on large-scale reactions to synthesize medi-MOF-1 toward the capture of I_2_ and gas sorption. We utilized medi-MOF-1 based on curcumin, which displays an interesting and important ability to capture I_2_ (1.936 g g^−1^) at room temperature. The medi-MOF-1 microcrystals have lower Langmuir specific surface areas than previously published medi-MOF-1. In particular, it is amongst the more efficient porous materials for I_2_ adsorption. Furthermore, medi-MOF-1 also exhibits hydrogen adsorption capacities of 1.57 wt.% at 77 K and 1 bar. The adsorption capacity of CO_2_ by activated medi-MOF-1 is greater than that of CH_4_ and N_2_ at 273 K, and the selectivities of CO_2_/N_2_ and CO_2_/CH_4_ are 5.8 and 2.5, respectively. This study aims to provide material for the potential application of I_2_ adsorption as well as gas adsorption and separations.

## Figures and Tables

**Table 1:** CO_2_ adsorption data of MOFs for some reported bio-MOFs at 77 K, 1 bar.

Materials	BET (m^2^ g^−1^)	Pore Volume (c g^−1^)	CO_2_ @273 K (cm^3^ g^−1^)	Ref.
bio-MOF-1	1680	0.75	76	[49]
bio-MOF-11	1148	0.45	147	[53]
bio-MOF-12	1008	0.42	100	[53]
bio-MOF-13	412	0.20	60	[53]
bio-MOF-14	17	0.035	45	[53]
medi-MOF-1	475	-	34.7	This work

**Table 2:** Iodine adsorption capacities in MOFs via vapor diffusion.

Materials	BET (m^2^ g^−1^)	Pore Volume (cm^3^ g^−1^)	Iodine Uptake (wt.%)	Ref.
Cu-BTC	1850	0.74	175	[40]
ZIF-8	1630	0.66	125	[54]
(ZnI_2_)_3_(tpt)_2_	-	-	173	[56]
Zr_6_O_4_(OH)_4_(sdc)_6_	2900	1.33	107	[59]
Zr_6_O_4_(OH)_4_(peb)_6_	2650	1.16	279	[59]
medi-MOF-1	475	-	193	This work

## Data Availability

No new data were created or analyzed in this study in this study. Data sharing is not applicable to this article.

## Supplementary Materials

The following supporting information can be downloaded at: https://www.mdpi.com/article/10.3390/molecules28135237/s1, Figure S1: The fitting data for calculating the H2 Qst value for medi-MOF-1; Figure S2: FT-IR of medi-MOF-1 and medi-MOF-1@I2; Figure S3: Up: visual color change of iodine solution in ethanol I2 adsorption progress of medi-MOF-1, Below: Photographs showing the color change iodine capture for medi-MOF-1; Table S1: Summary of H2 uptake and Q_st_ value for some reported MOFs at 77K, 1bar.
