# Supplementary material for: Study on Gas Sorption and Iodine Uptake of a Metal-Organic Framework Based on Curcumin
Source: Molecules. 2023 Jul 6;28(13):5237. doi: 10.3390/molecules28135237 (PMC10343388; doi:10.3390/molecules28135237)
Supplement: Supplementary file 1 [file molecules-28-05237-s001.zip › molecules-2453885-supplementary.pdf]

molecules

MDPI

Supplementary Materials

Study on Gas Sorption and Iodine Uptake of a Metal–Organic Framework Based on Curcumin

Hongmin Su [1], Yang Zhou [1], Tao Huang [1] and Fuxing Sun [2,*]

[1] School of Environmental Science and Engineering, Yancheng Institute of Technology, Yancheng 224051, China
[2] State Key Laboratory of Inorganic Synthesis and Preparative Chemistry, College of Chemistry, Jilin University, Changchun 130021, China
* Correspondence: fxsun@jlu.edu.cn

Isosteric Heat of Adsorption

The binding energy between host material and guest molecules is reflected in the isosteric heat of adsorption. The isosteric heats of adsorption (Q_{st}) for each gas were obtained on the basis of the Clausius-Clapeyron equation, which could be defined as:

$$Q_{st} = RT^2\left(\frac{\partial \ln P}{\partial T}\right)_q$$

Here, Q_{st} is the coverage-dependent isosteric heat of adsorption, R is the universal gas constant, P is the pressure, q is the amount adsorbed, T is the temperature.

The experimental isotherms at 273 and 298 K were fitting by a virial-type expression of the following form:

$$\ln P = \ln N + \frac{1}{T}\sum_{i=0}^{m} a_i N^i + \sum_{i=0}^{n} b_i N^i$$

Here, P is the pressure expressed in bar, N is the amount adsorbed in mmol/g, T is the temperature in K, ai and bi are virial coefficients, and m and n represent the number of coefficients required to adequately describe the isotherms. The equation was fit using the R statistical software package; m and n were gradually increased until the contribution of extra added a and b coefficients was deemed to be statistically insignificant toward the overall fit, as determined using the t-test. The values of the virial coefficients a_0 through a_m were then used to calculate the isosteric heat of adsorption using the following expression:

$$-Q_{st} = -R\sum_{i=0}^{m} a_i N^i$$

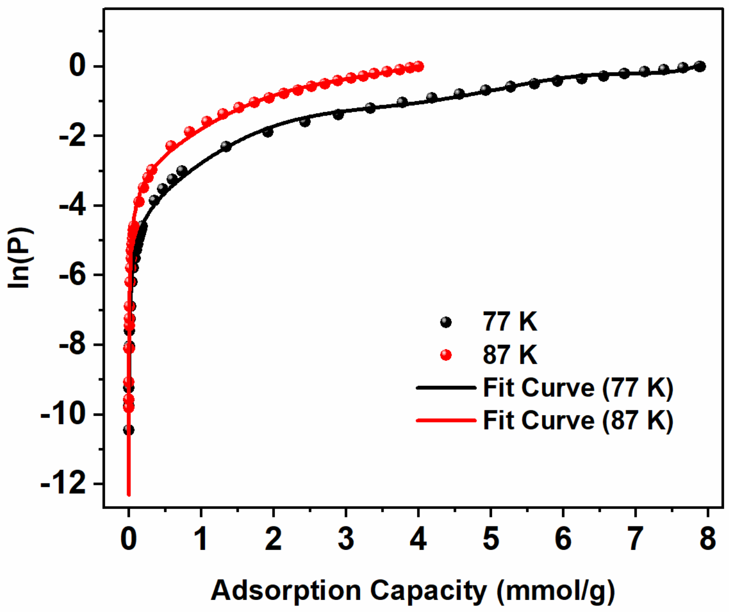

Figure S1 The fitting data for calculating the H_2 Q_{st} value for medi-MOF-1

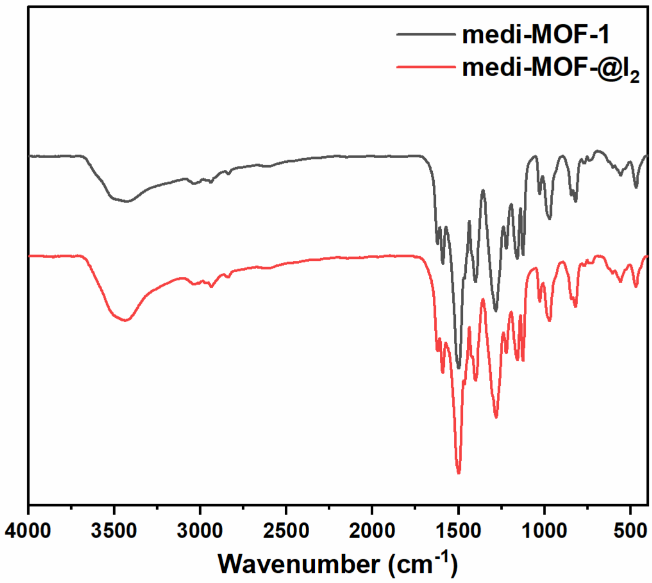

Figure S2 FT-IR of medi-MOF-1 and medi-MOF-1@I_2

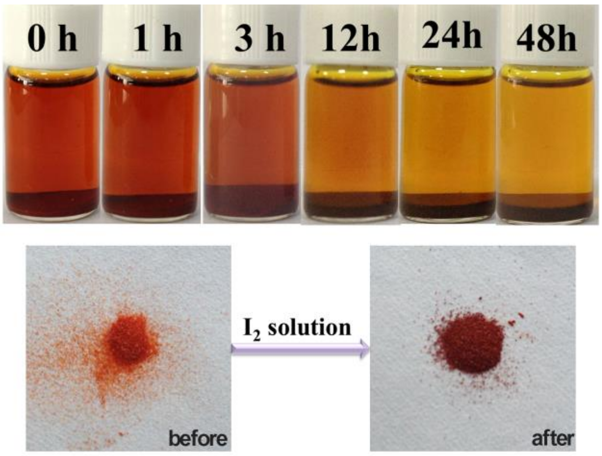

Figure S3 Up: visual color change of iodine solution in ethanol I_2 adsorption progress of medi-MOF-1; Below: Photographs showing the color change iodine capture for medi-MOF-1.

Table S1. Summary of H_2 uptake and Q_{st} value for some reported MOFs at 77 K, 1 bar

Materials	BET ($m^2\ g^{-1}$)	Langmuir surface area ($m^2\ g^{-1}$)	Q_{st} H_2 ($kJ\ mol^{-1}$)	Ref.
bio-MOF-11	1148	-	-13	[47]
M_2(olz)(Mg)	2545	4593	-10.8	[48]
M_2(olz)(Fe)	1485	2618	-10.9	[48]
M_2(olz)(Co)	2060	3838	-11.4	[48]
M_2(olz)(Ni)	2067	3813	-12.1	[48]
M_2(olz)(Zn)	636	770	-7.3	[48]
medi-MOF-1	475	563	-6.25	This work
